# Data on the design of truss structures by teams of engineering students

**DOI:** 10.1016/j.dib.2018.02.078

**Published:** 2018-03-08

**Authors:** Christopher McComb, Jonathan Cagan, Kenneth Kotovsky

**Affiliations:** a School of Engineering Design, Technology, and Professional Programs, Pennsylvania State University, University Park, PA, USA; b Department of Mechanical Engineering, Carnegie Mellon University, Pittsburgh, PA, USA; c Department of Psychology, Carnegie Mellon University, PA, USA

**Keywords:** Engineering, Design, Teams, Structures

## Abstract

This experiment was conducted in order to compare different approaches that human teams use to solve design problems that change dynamically during solving. Specifically, study participants were given the task of designing a truss structure (similar to a bridge spanning a chasm) in teams of three. At two points during design, the problem statement was changed unexpectedly, requiring participants to adapt. Two conditions were given different initial problem representations. During the study, every participant had access to a computer interface that allowed them to construct, test, and share solutions. The interface also made it possible to collect a step-by-step log of the actions made by participants during the study. This article contains data collected from 48 participants (16 teams). This data has been used previously in behavioral analyses, sequence-based analysis, and development of computational models.

## Specifications Table

**Table:** 

Subject area	*Engineering, Design*
More specific subject area	*Configuration design by engineering students*
Type of data	*Table*
How data was acquired	*Desktop computer*
Data format	*Raw data*
Experimental factors	*Conditions: (1) control condition with basic problem statement, (2) experimental condition with extended problem statement.*
Experimental features	*Engineering students designed a truss in the conditions noted above. Design was facilitated through a computer interface*
Data source location	*Pittsburgh, PA*
Data accessibility	*Data is available as a supplementary attachment to this article.*

## Value of the data

• The information here is important to engineering design since it provides a step-by-step history of problem-solving in engineering design.
• Truss design is a common task used to benchmark computational design algorithms, and this dataset can serve as an external comparison in those efforts.
• Researchers in cognitive science may be interested in this data set, since it provides a contextual lens through which to study problem solving. Often the challenge in such studies is an analysis of progress to goals; this work includes a progress metric for each problem solving step.

## 1 Data

This dataset is provided as supplementary data in a CSV format. Each row in the CSV describes a component of a truss design produced in the study. Descriptions of the columns headings are provided in Table 1.

**Table 1:** Description of columns in attached data table.

**Column header**	**Description**
Condition	An integer indicating the condition of the individual who produced the design. A value of 1 indicates the control condition, and a value of 2 indicates the experimental condition.
Team	An integer indicating the team identification (1–16)
Performance Echelon	An integer indicating 5 highest-performing teams (+ 1) 5 lowest-performing teams (− 1) and other teams (0). Echelons based on post-hoc analysis from previous work.
Participant	An integer identifying the participant on the team (1–3)
Session	An integer identifying which 4-min design session (1–6) the design was created during.
Design	An integer identifying different designs.
Mass	The mass of the design in kilograms.
FOS1	The factor of safety of the design with respect to the first problem statement.
FOS2	The factor of safety of the design with respect to the second problem statement.
FOS3	The factor of safety of the design with respect to the third problem statement.
Component ID	An integer identifying different components within the design. Positive values indicate joints, negative values indicate members
	
Var1	For joints: location along the *x*-axis in meters.
	For members: component integer identifying first joint connected to.
	
Var2	For joints: location along the *y*-axis in meters.
	For members: component integer identifying second joint connected to.
	
Var3	For joints: number of degrees-of-freedom that are restricted. A value of 0 indicates constraints on motion in both *x* and *y* directions. A value of 1 indicates a constraint on motion in the *y* direction only. A value of 0 indicates no constraints.
	For members: An integer indicating the size of the member. Detailed size information is given in Table 2.

## 2 Experimental design, materials, and methods

### 2.1 Participants and conditions

This study was conducted with senior undergraduate students in mechanical engineering. Participants in the study were placed in one of two conditions. Participants in the control condition were given the task of designing a truss structure based on a simple problem statement. Participants in the experimental condition were provided with an extended problem statement that tasked them with examining additional loading scenarios for the truss. The exact text of each problem statement is provided in other work [1].

### 2.2 Design task

Participants were specifically tasked with designing a truss to span a chasm. They were required to use three support joints and two loading joints that were provided. Thus, these five joints were present in every design. The process of designing a truss consisted of placing joints and connecting them with structural members of varying size. The available sizes for structural members are provided in Table 2. Only cylindrical tubes for structural members were permitted.

**Table 2:** Member properties for different member sizes (Var3 in Supplementary data table).

**Member size**	**Outer diameter (cm)**	**Inner diameter (cm)**	**Cross-sectional area (cm**^**2**^**)**	**Moment of inertia (cm**^**4**^**)**
1	1.000	0.867	0.195	0.021
2	2.000	1.733	0.782	0.342
3	3.000	2.600	1.759	1.733
4	4.000	3.467	3.128	5.477
5	5.000	4.333	4.887	13.371
6	6.000	5.200	7.037	27.726
7	7.000	6.067	9.578	51.367
8	8.000	6.933	12.511	87.629
9	9.000	7.800	15.834	140.365
10	10.000	8.667	19.548	213.939

Design took place over six four-minute design sessions. Participants were subjected to two unexpected changes to their problem statement. Initially, they were instructed to design a truss with a mass of 175 kg or less and a factor-of-safety of at least 1.25 (e.g., strength at least 1.25 times the expected load). The first change was provided after the end of the third design session and required participants to design a truss that would remain stable even if one support joint was removed. The factor-of-safety for this new case was relaxed to 1.00, with a new target mass of 350 kg. The second change was provided after the end of the fourth design session, and tasked participants with designing their truss to pass around an obstacle. The required factor-of-safety was returned to 1.25 and the mass target was set at 200 kg. Additional information about these changes is provided in other work [1].

### 2.3 Design interface

Study participants were provided with a graphical user interface. This interface made it possible for them to construct truss designs, evaluate the mass and factor-of-safety of the designs, and share them within their team. The sharing mechanism enabled participants to adopt the design of one of their teammates at any time, replacing the design of the adopter.

This interface was also used to capture step-by-step data for each of the participants, creating the dataset shared here. This data is rich enough to facilitate detailed behavioral analysis [1], design process analysis [2], [3], and comparison to the output of computational team simulations using the Cognitively-Inspired Simulated Annealing Teams (CISAT) modeling framework [4].
